# Cisplatin-Resistant CD44^+^ Lung Cancer Cells Are Sensitive to Auger Electrons

**DOI:** 10.3390/ijms23137131

**Published:** 2022-06-27

**Authors:** Karina Lindbøg Madsen, Oke Gerke, Poul F. Høilund-Carlsen, Birgitte Brinkmann Olsen

**Affiliations:** 1 Department of Nuclear Medicine, Odense University Hospital, DK-5000 Odense, Denmark; klma@ucl.dk (K.L.M.); oke.gerke@rsyd.dk (O.G.); pfhc@rsyd.dk (P.F.H.-C.); 2 Department of Clinical Research, University of Southern Denmark, DK-5000 Odense, Denmark

**Keywords:** auger electrons, lung cancer, CD44, cancer stem cells, cisplatin, DNA damage, apoptosis

## Abstract

Cancer stem cells (CSCs) are resistant to conventional therapy and present a major clinical challenge since they are responsible for the relapse of many cancers, including non-small cell lung cancer (NSCLC). Hence, future successful therapy should also eradicate CSCs. Auger electrons have demonstrated promising therapeutic potential and can induce DNA damage while sparing surrounding cells. Here, we sort primary patient-derived NSCLC cells based on their expression of the CSC-marker CD44 and investigate the effects of cisplatin and a thymidine analog (deoxyuridine) labeled with an Auger electron emitter (^125^I). We show that the CD44^+^ populations are more resistant to cisplatin than the CD44^−^ populations. Interestingly, incubation with the thymidine analog 5-[^125^I]iodo-2′-deoxyuridine ([^125^I]I-UdR) induces equal DNA damage, G2/M cell cycle arrest, and apoptosis in the CD44^−^ and CD44^+^ populations. Our results suggest that Auger electron emitters can also eradicate resistant lung cancer CD44^+^ populations.

## 1. Introduction

Lung cancer remains the leading cause of cancer mortality, with an estimated 2.2 million cases newly diagnosed and 1.8 million deaths globally in 2020 [1]. Traditionally, lung cancer is classified by histology as small cell lung cancer (SCLC) or non-small cell lung cancer (NSCLC), the latter of which is further divided into squamous cell carcinoma, adenocarcinoma, and large cell carcinoma [2]. NSCLC is the predominant type of lung cancer, and the 5-year survival rate is ~20% [1,3]. Most lung cancer patients do not undergo surgery due to late diagnosis and advanced progression. Advanced NSCLC is treated with chemotherapy, radiotherapy, targeted therapy, and immunotherapy; however, resistance leads to relapse (reviewed in [4,5,6]). The presence of subpopulations within tumors, consisting of so-called cancer stem cells (CSCs), is thought to be responsible for the development of resistance. CSCs can self-renew (i.e., form spheres), differentiate, and metastasize [7,8,9] and have been discovered in many cancers, including brain, liver, breast, gastric, prostate, and lung cancer (reviewed in [10,11]). They are resistant to conventional therapy due to slow proliferation, changes in the expression of cell cycle-related proteins [12], increased DNA repair capability [13], the increased activation of DNA damage checkpoint proteins [14], and the overexpression of anti-apoptotic proteins, efflux transporters, and ROS (reactive oxygen species) scavengers [15]. Furthermore, CSCs are characterized by their expression of surface markers, including (cluster of differentiation) CD44, CD90, and CD133 [16]. CD44 is a transmembrane glycoprotein expressed on CSCs in several cancers and implicated in tumor development, invasion, and metastasis [17,18]. In addition, the overexpression of CD44 has been associated with poor prognosis [19] and drug resistance [20] in lung cancer. However, not all CSCs express the same surface markers, and CSCs of the same origin can express different surface markers. This also means that isolation based on surface markers is an enrichment of CSCs [21].

Auger electron emitters could be a promising approach to overcome treatment resistance in lung cancer as they have shown therapeutic potential in breast cancer, multiple myeloma, and brain cancer [22,23,24,25]. Auger electrons are ejected by radionuclides decaying by electron capture and/or internal conversion. The electrons have potential for targeted therapy due to their nanometer range and high linear energy transfer (LET) of 4 to 26 keV/μm [26]. Auger electron emitters incorporated into the DNA are 10–100-fold more toxic than when the decay occurs in the cytosol or extracellular space [25,26]. The Auger-emitting thymidine analog 5-[^125^I]iodo-2′-deoxyuridine ([^125^I]I-UdR) is incorporated into the DNA of proliferating cells, where it induces lethal DNA double-strand breaks (DSBs) [27,28]. DNA DSBs will initiate the DNA damage response; however, if the damage exceeds the repair capacity of the cell, the cell will undergo permanent cell cycle arrest or cell death [29].

In the present study, we sort patient-derived lung cancer cells based on their expression of CD44 and show that CD44^+^ cells are resistant to cisplatin. In addition, we show that the thymidine analog [^125^I]I-UdR could overcome resistance, since its cellular responses (viability, DNA DSBs, cell cycle arrest, and apoptosis) are equal in the sorted subpopulations.

## 2. Results

The patient-derived cells were grown as free-floating tumorspheres in serum-free medium to enrich stemness features, including unlimited growth abilities, tumorigenic potential in vivo, ability to differentiate, high invasion capacity, and resistance to chemotherapy [30,31]. Magnetic-labeled CD44 antibodies were used to sort cells, and freshly sorted CD44-positive and CD44-negative populations were used for experiments.

### 2.1. Tumorsphere Formation and qPCR

**Figure 1:**
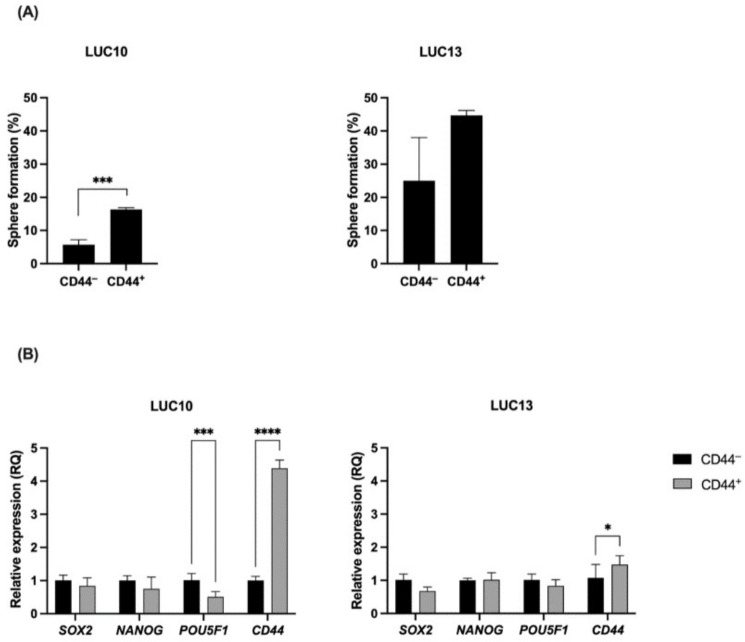
Tumorsphere formation and the relative expression of *SOX2, NANOG, POU5F1,* and *CD44*. (**A**) Generation of tumorspheres from a single cell. (**B**) Relative expression of pluripotency markers *SOX2, NANOG,* and *POU5F1* and the surface marker *CD44* in sorted LUC10 and LUC13 cells. Experiments were performed as three independent replicates. Values are expressed as the mean ± standard deviation. *: *p <* 0.05; ***: *p <* 0.001; and ****: *p <* 0.0001.

As CD44 is one of the most commonly used surface markers to identify lung CSCs [32], we initially investigated the ability of freshly sorted CD44^−^ and CD44^+^ cells to form spheres and their expression of pluripotency genes. Using previously characterized patient-derived cell lines LUC10 and LUC13 [33], we found that the tumorsphere formation ability of CD44^+^ LUC10 cells was significantly higher (*p <* 0.001) compared to CD44^−^ LUC10 cells. For LUC13 cells, the difference was not significant, although there was a tendency for higher sphere formation in the CD44^+^ cells than in the negative cells (Figure 1A).

The relative expression levels of *SOX2*, *NANOG*, and *POU5F1* (Oct4), transcription factors essential for pluripotency maintenance and self-renewal, and the CSC marker *CD44*, were evaluated in freshly sorted LUC10 and LUC13 cells (Figure 1B). *SOX2* and *NANOG* were not differentially expressed in the sorted cell populations, whereas POU5F1 was significantly (*p <* 0.001) less expressed in LUC10 CD44^+^ than CD44^−^ cells. The relative expression of CD44 was significantly (*p <* 0.0001) increased in the CD44^+^ LUC10 population compared to the CD44^−^ population. Additionally, CD44^+^ LUC13 expressed significantly (*p <* 0.05) more CD44 than the CD44^−^ population, although the difference was not as pronounced as LUC10. Overall, these results indicate that CD44^+^ cells are more likely to form tumorspheres; however, the pluripotency genes were not upregulated in the CD44^+^ cells.

### 2.2. Doubling Time and Proliferation

**Figure 2:**
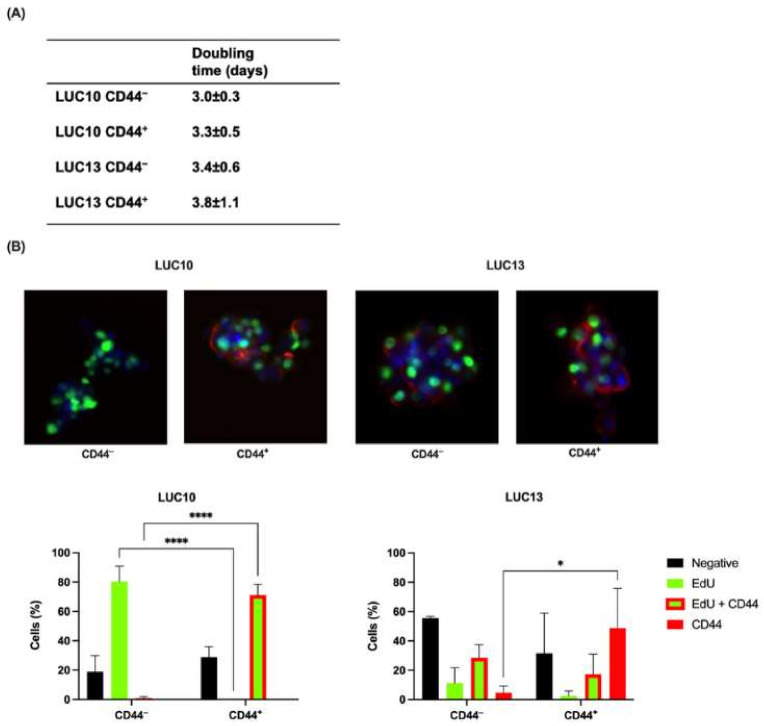
Doubling time and proliferation. (**A**) The doubling time for CD44-sorted cells grown as tumorspheres. (**B**) Upper panel: representative fluorescence microscopy images of CD44-sorted cells labeled with EdU (proliferation; green), PE-labeled anti-CD44 antibodies (red), and counterstained with DAPI (blue; magnification ×20). Lower panel: quantification of negative, EdU-positive, EdU- and CD44-positive, and CD44-positive cells. Experiments were performed as three independent replicates. Values are expressed as the mean ± standard deviation. *: *p <* 0.05 and ****: *p <* 0.0001.

Next, we characterized the sorted populations with respect to doubling time and proliferation. Although not significant, the doubling time was slightly longer for CD44^+^ cells than for CD44^−^ cells (Figure 2A). Furthermore, the doubling time was nearly identical for LUC10 and LUC13.

The thymidine analog EdU was used to determine the proliferation of the sorted cells, and this was combined with CD44 immunostaining (Figure 2B). Most of the CD44^−^ LUC10 cells were CD44-negative and only EdU-positive, i.e., proliferating. This was significantly (*p <* 0.0001) higher than CD44^+^ since the majority of the CD44^+^ LUC10 cells were CD44-positive, a significant (*p <* 0.0001) increase compared to CD44^−^. However, all the CD44^+^ cells were also proliferating, i.e., EdU-positive. The majority of CD44^−^ LUC13 cells were negative for EdU and CD44. One third were both EdU- and CD44-positive, and the remaining cells were either proliferating or CD44-positive. Half of the CD44^+^ LUC13 cells were positive for CD44, significantly higher (*p <* 0.05) than the CD44^−^ population. Furthermore, almost one third of cells were negative for CD44 and EdU, and 20% of the CD44^+^ were CD44-positive and proliferating.

Overall, these results suggest that the sorting was more efficient in LUC10, since there were CD44^+^ and CD44^−^ cells in both LUC13 populations.

### 2.3. Cisplatin

**Figure 3:**
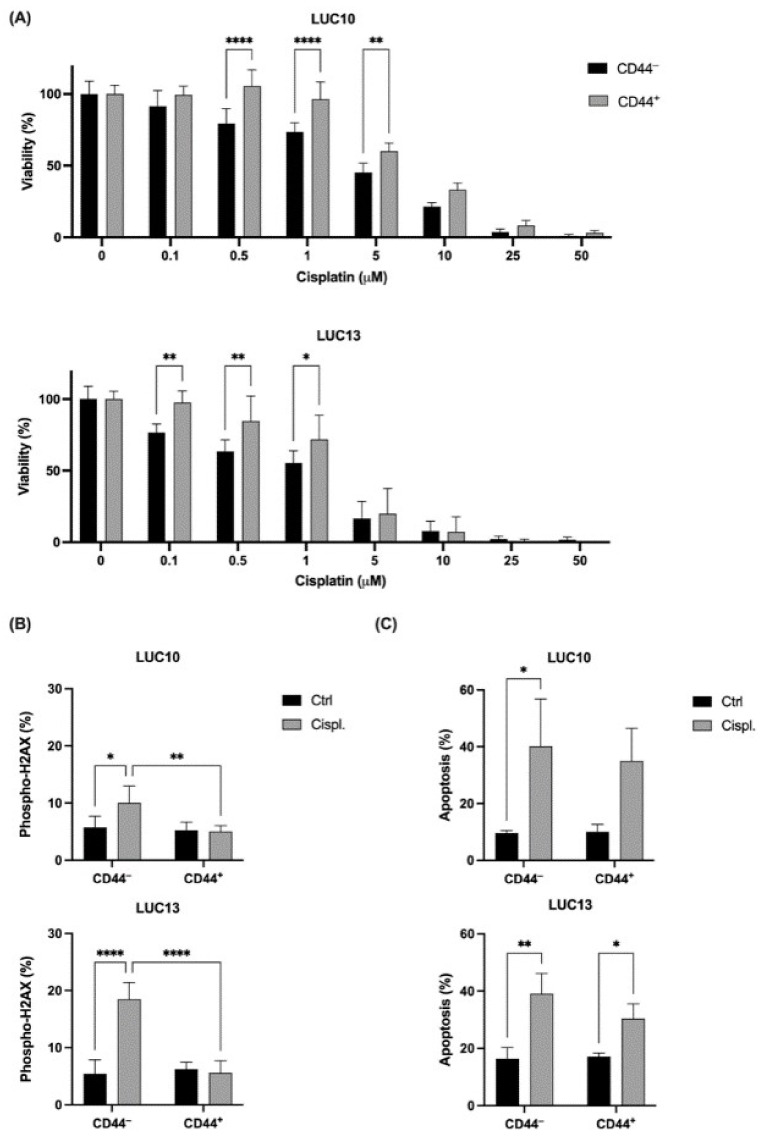
Viability, DNA damage, and apoptosis (annexin V-positive cells) after cisplatin treatment. (**A**) The viability was evaluated using the CellTiter-Blue assay after 72 h incubation with increasing concentrations of cisplatin. (**B**) The percentage of DNA DSBs after treatment with 10 μM cisplatin for 2 h and (**C**) apoptosis after 72 h. Experiments were performed as three independent replicates. Values are expressed as the mean ± standard deviation. *: *p <* 0.05; **: *p <* 0.01; and ****: *p <* 0.0001.

One of the hallmarks of CSCs is their resistance to chemotherapeutic drugs. Since cisplatin is the first-line chemotherapy against advanced NSCLC, we tested the viability in response to 0.1–50 μM cisplatin for 72 h (Figure 3A). There was a significant dose-dependent decrease in the viability of CD44^−^ LUC10 cells at ≥ 0.5 μM cisplatin (*p <* 0.0001) compared to untreated control cells. The viability of CD44^+^ LUC10 cells also decreased significantly, but only ≥5 μM cisplatin (*p <* 0.0001) compared to the untreated control cells. Hence, the viability of CD44^+^ LUC10 cells was significantly higher at 0.5, 1 (*p <* 0.0001), and 5 μM cisplatin (*p <* 0.01) than that of the CD44^−^ cells. There was also a significant reduction in cell viability for the CD44^−^ LUC13 cells at all cisplatin concentrations (*p <* 0.01 for 0.1 μM and *p <* 0.0001 for 0.5–10 μM) compared to the untreated control cells. In contrast, the viability of the CD44^+^ LUC13 cells was only significantly reduced at concentrations of ≥ 1 μM (*p <* 0.0001) compared to the untreated control cells. The difference in viability between the CD44^−^ and CD44^+^ LUC13 cells was significant at 0.1, 0.5 (*p <* 0.01), and 1 μM (*p <* 0.05).

The phosphorylation of H2AX (phospho-H2AX) is a marker of DNA DSBs [34] and was examined after treatment with 10 μM cisplatin for 2 h (Figure 3B). The level of phospho-H2AX increased significantly in the cisplatin-treated CD44^−^ LUC10 cells (*p <* 0.05). In contrast, there was no induction of phospho-H2AX in the CD44^+^ LUC10 cells, which gave a significant (*p <* 0.01) difference in phospho-H2AX between the two populations. Similarly, cisplatin-induced H2AX phosphorylation was only present in the CD44^−^ LUC13 cells (*p <* 0.0001); hence, there was significantly (*p <* 0.0001) more phospho-H2AX in the CD44^−^ LUC13 cells compared to the CD44^+^ LUC13 cells. If radiation-induced damage is left unrepaired, it can lead to apoptosis; hence, annexin V-positive cells were evaluated following treatment with 10 μM cisplatin for 72 h (Figure 3C). Cisplatin increased the percentage of annexin V-positive CD44^−^ LUC10 (*p <* 0.05) cells compared to untreated cells. There was also an increase in CD44^+^ cells, although not significant. For LUC13, cisplatin significantly increased annexin V-positive CD44^−^ cells (*p <* 0.01) and also CD44^+^ cells (*p <* 0.05).

Overall, the CD44^+^ cells from LUC10 and LUC13 were more resistant to cisplatin, as measured by viability, and exhibited less DNA damage than the CD44^−^ cells. However, we could not detect any significant difference in apoptosis induction.

### 2.4. Cellular Uptake and DNA Incorporation of [^125^I]I-UdR

**Figure 4:**
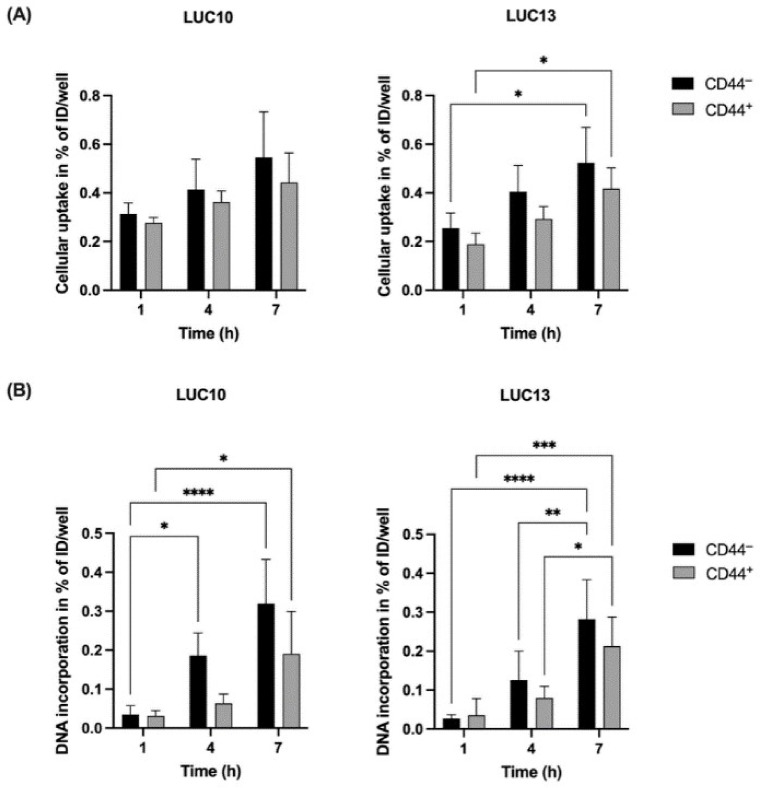
Cellular uptake and DNA incorporation of [^125^I]I-UdR. (**A**) Cellular uptake of 18.5 kBq/mL [^125^I]I-UdR after 1, 4, and 7 h. (**B**) DNA incorporation of 18.5 kBq/mL [^125^I]I-UdR after 1, 4 and 7 h. Experiments were performed as three independent replicates. Values are expressed as the mean ± standard deviation. *: *p <* 0.05; **: *p <* 0.01; ***: *p <* 0.001; and ****: *p <* 0.0001.

DNA incorporation is necessary for the Auger electron-emitting thymidine analog [^125^I]I-UdR to effectively induce DNA damage [27]. Therefore, we investigated the cellular uptake and DNA incorporation of [^125^I]I-UdR at 1, 4, and 7 h in the freshly sorted LUC10 and LUC13 cells. The cellular uptake of [^125^I]I-UdR in both LUC10 subpopulations increased over time; however, not significantly (Figure 4A). The cellular uptake in LUC13 cells increased significantly between 1 and 7 h for both subpopulations (*p <* 0.05). The DNA incorporation of [^125^I]I-UdR increased significantly between 1 and 4 h (*p <* 0.05) and 1 and 7 h (*p <* 0.0001) in CD44^−^ LUC10. The increase was only significant after 7 h (*p <* 0.05) in CD44^+^ LUC10 (Figure 4B). For CD44^−^ LUC13, the DNA incorporation increased significantly between 1 and 7 h (*p <* 0.0001) and between 4 and 7 h (*p <* 0.01). For CD44^+^ LUC13, there was also a significant increase between 1 and 7 h (*p <* 0.001) and between 4 and 7 h (*p <* 0.05) (Figure 4B). For LUC13 and LUC10, there were no significant differences between the CD44^−^ and CD44^+^, although uptake and DNA incorporation were consistently lower in the CD44^+^ cells.

### 2.5. Viability

**Figure 5:**
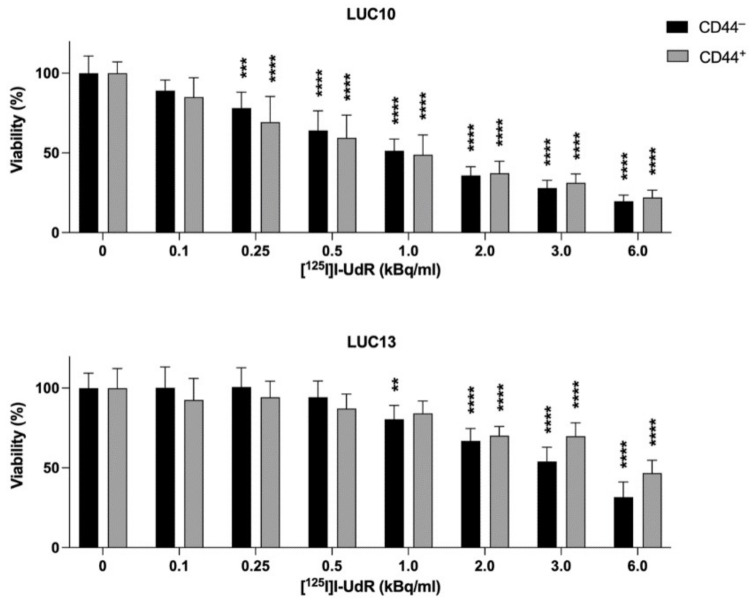
Cell viability after [^125^I]I-UdR treatment. The viability was evaluated using the CellTiter-Blue assay after 7 days of incubation with increasing activity concentrations of [^125^I]I-UdR. Experiments were performed as three independent replicates. Values are expressed as the mean ± standard deviation. **: *p <* 0.01; ***: *p <* 0.001; and ****: *p <* 0.0001.

Next, we tested the effect of [^125^I]I-UdR on the viability of the freshly sorted cells by incubating with 0.1–6.0 kBq/mL [^125^I]I-UdR for 7 days (Figure 5). For CD44^−^ LUC10, there was a significant dose-dependent decrease in viability (0.25 kBq/mL: *p <* 0.001; and 0.5–6 kBq/mL: *p <* 0.0001) when treated with doses ≥ 0.25 kBq/mL [^125^I]I-UdR compared to the untreated control cells. The same decrease in viability was also observed for CD44^+^ LUC10 (*p <* 0.0001). However, there was no significant difference between CD44^−^ LUC10 and CD44^+^ LUC10 at any activity concentration. CD44^−^ LUC13 showed a significant dose-dependent decrease in viability when treated with doses ≥ 1 kBq/mL (1 kBq/mL: *p <* 0.01 and 2–6 kBq/mL: *p <* 0.0001). The viability of CD44^+^ LUC13 was significantly decreased ≥ 2 kBq/mL (*p <* 0.0001). Differences between CD44^−^ LUC13 and CD44^+^ LUC13 were not significant. The non-radioactive but chemically identical [^127^I]I-UdR did not affect the viability (data not shown).

Overall, the LUC10 cells appeared more sensitive than LUC13; however, the CD44^+^ and CD44^−^ cells were equally sensitive to Auger electrons, indicating that the Auger electrons could overcome the resistance observed for cisplatin.

### 2.6. DNA Damage

**Figure 6:**
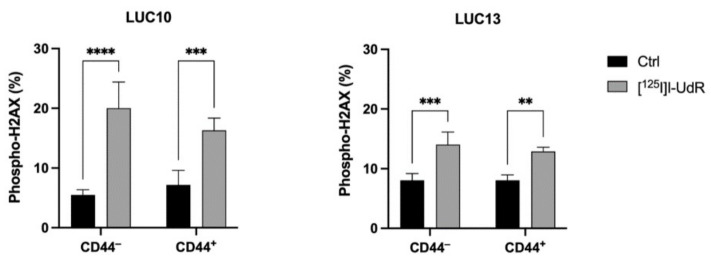
Radiation-induced DNA damage. The percentage of DNA DSBs after treatment with 2.5 kBq/mL [^125^I]I-UdR was evaluated by the analysis of phospho-H2AX after 7 days. Experiments were performed as three independent replicates. Values are expressed as the mean ± standard deviation. **: *p <* 0.01; ***: *p <* 0.001; and ****: *p <* 0.0001.

To further understand the effects of the Auger electrons, we analyzed DNA DSBs by measuring the percentage of phospho-H2AX after treatment with 2.5 kBq/mL [^125^I]I-UdR for 7 days (Figure 6). [^125^I]I-UdR induced a significant amount of DNA DSBs in both CD44^−^ and CD44^+^ LUC10 cells (CD44^−^: *p <* 0.0001 and CD44^+^: *p <* 0.001). Phospho-H2AX foci in CD44^−^ and CD44^+^ LUC13 also increased significantly (CD44^−^: *p <* 0.001; CD44^+^: *p <* 0.01). Unlike cisplatin, [^125^I]I-UdR induced similar amounts of DNA DSBs in the CD44^−^ and CD44^+^ populations.

### 2.7. Cell Cycle

**Figure 7:**
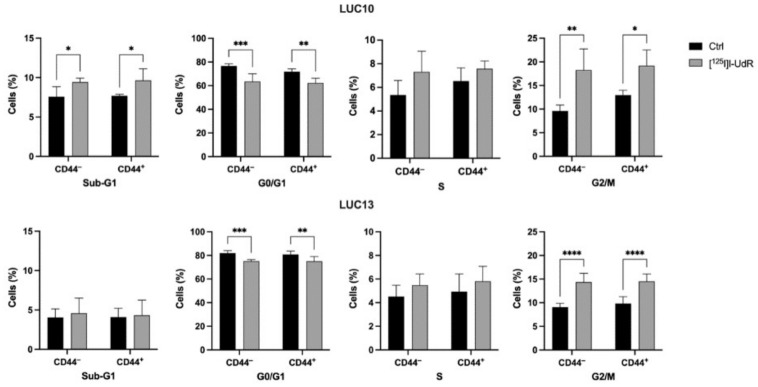
Radiation-induced changes in the cell cycle. Cell cycle analysis after treatment with 2.5 kBq/mL [^125^I]I-UdR for 7 days. Experiments were performed as three independent replicates. Values are expressed as the mean ± standard deviation.*: *p <* 0.05; **: *p <* 0.01; ***: *p <* 0.001; and ****: *p <* 0.0001.

Since the Auger electrons induced DNA DSBs, we analyzed the cell cycle to detect any changes compared to untreated control cells. The effects in the CD44-sorted LUC10 and LUC13 were analyzed by treatment with 2.5 kBq/mL [^125^I]I-UdR for 7 days (Figure 7). In LUC10, [^125^I]I-UdR significantly increased the percentage of CD44^−^ and CD44^+^ in subG1 (*p <* 0.05) and G2/M (CD44^−^: *p <* 0.01; CD44^+^: *p <* 0.05) Concomitantly, cells in G0/G1 decreased significantly (CD44^−^: *p <* 0.001; CD44^+^: *p <* 0.01). Unlike LUC10, [^125^I]I-UdR treatment did not increase sub-G1 in LUC13 but led to a significant increase in G2/M for both CD44 populations (*p <* 0.0001). At the same time, the proportion of LUC13 in G0/G1 significantly decreased (CD44^−^: *p <* 0.001; CD44^+^: *p <* 0.01). These results suggest that [^125^I]I-UdR induced a similar G2/M arrest regardless of CD44 expression.

### 2.8. Annexin

**Figure 8:**
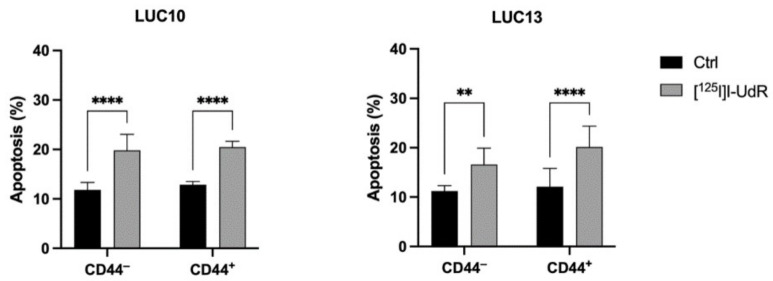
Radiation-induced apoptosis (annexin V-positive cells). Apoptosis after treatment with 2.5 kBq/mL [^125^I]I-UdR evaluated by annexin V after 7 days. Experiments were performed as three independent replicates. Values are expressed as the mean ± standard deviation. **: *p <* 0.01; ****: *p <* 0.0001.

Finally, we evaluated apoptosis (annexin V-positive cells) after treatment with 2.5 kBq/mL [^125^I]I-UdR for 7 days (Figure 8). Treatment with [^125^I]I-UdR significantly increased annexin-V positive CD44^−^ and CD44^+^ LUC10 cells (*p <* 0.0001). [^125^I]I-UdR also significantly increased annexin V-positive CD44^−^ and CD44^+^ LUC13 cells (*p <* 0.01 and *p <* 0.0001, respectively). The lack of difference in apoptosis induction between the populations supports the notion that Auger electrons also target CD44^+^ cells.

## 3. Discussion

Although new treatments are continuously developed, the management of lung cancer remains a major clinical challenge. Here, we investigated the effects of a thymidine analog labeled with an Auger electron emitter in CD44-sorted primary patient-derived lung tumor cells. The Auger electron-emitting compound [^125^I]I-UdR has previously proved effective in CSCs from glioblastoma, multiple myeloma, and lung cancer [23,25,33,35,36]. Here, we further investigated the effects in freshly sorted lung cancer cells and found that the CD44^+^ population was more resistant to cisplatin than the CD44^−^ population. However, CD44^−^ and CD44^+^ were equally sensitive to treatment with [^125^I]I-UdR, i.e., [^125^I]I-UdR could overcome the resistance of both populations.

The CD44^+^ population had a higher sphere-forming ability, indicating that CD44^+^ cells could self-renew, a hallmark of CSCs [17,18]. However, we could not detect an increased expression of the stemness transcription factors *SOX2*, *NANOG*, and *POU5F1* in the CD44^+^ populations. This is in contrast to other authors, who found an increased expression in CD44^+^ NSCLC cells compared to CD44^−^ cells [17,37]. However, they did not grow their cells as tumorspheres, prior to sorting, and the expression of pluripotency factors was much higher than adherent cells since tumorsphere culture is an enrichment of CSCs [38]. We have also previously found that the expression of, particularly, NANOG and SOX2 was increased when LUC10 and LUC13 were grown in serum-free medium compared to adherent cell culture in the presence of serum [33]. Furthermore, we only sorted based on CD44 expression, one of several putative lung CSC-markers. Almost certainly, other CSC subpopulations are present in the CD44^−^ and CD44^+^ populations, characterized by different CSC surface markers, i.e., CD133 or CD90 [16].

As expected, CD44 was expressed at higher levels in the CD44^+^ populations. However, a high percentage of CD44-positive cells was also present in the CD44^−^ LUC13 population, indicating that MACS was insufficient for complete sorting. LUC10 was more efficiently sorted than LUC13, which could be due to the expression of CD44 being more strongly associated with the squamous cell carcinoma subtype than adenocarcinoma [39]. The lower purity of the LUC13-sorted populations could be due to heterogeneous CD44 expression, e.g., cells with lower CD44 expression could be more challenging to retain to the column, and they would be present in the CD44^−^ population. We also cannot exclude that LUC13 cells aggregated, despite filtration, prior to loading, which may contribute to insufficient sorting [40]. It is also worth noticing that the staining in Figure 2B was performed 24 h after sorting, and it has been shown in prostate cancer cell lines that sorted CD44^−^ cells could convert to CD44^+^ cells [41]. We found that a large percentage of the CD44^+^ LUC10 population was also EdU-positive, which agrees with previous results that showed that CD44 could promote the cell proliferation of NSCLC cells [42]. However, Alowaidi et al. [43] found that cells expressing CD44 had lower EdU incorporation, indicating that the role of CD44 in NSCLC requires further studies.

The CD44^+^ cells were more resistant to cisplatin than CD44^−^ cells, as also found by Leung et al. [17]. We only observed this at lower concentrations, whereas there were no significant differences in the viability assays at higher concentrations. Yin et al. found that the silencing of CD44 improved sensitivity to cisplatin, and high cisplatin concentrations affected the viability equally in cells regardless of CD44 expression [44]. Cisplatin only induced DNA DSBs in the CD44^−^ cells, correlating with previous results, where cisplatin only induced DSBs in cisplatin-sensitive and not in resistant cells [45]. Phospho-H2AX is a marker of DSBs; thus, we cannot exclude that other types of damage were induced [46]. Other explanations for the lack of phospho-H2AX in the CD44^+^ cells include increased repair, since CSCs exhibit increased DNA repair capabilities [13] and cisplatin is exported via the increased expression of efflux proteins [47]. The cisplatin-dependent induction of apoptosis was almost equal in CD44^−^ and CD44^+^ cells. Leung et al. found a lower level of apoptosis in CD44^+^ lung cancer cells in response to cisplatin [17]. However, we used 10 µM cisplatin for the apoptosis assays where no resistance was observed in the viability assays, which supports the finding that resistance was found at lower concentrations [44]. No major differences were found for [^125^I]I-UdR uptake in the CD44^−^ and CD44^+^ cells. The incorporation was lower than previously found for unsorted LUC10 and LUC13 cells [33], which might be explained by decreased proliferation immediately after sorting [40].

Unlike for cisplatin, there was no difference in viability between the sorted populations in response to [^125^I]I-UdR. Furthermore, [^125^I]I-UdR induced the same degree of DNA DSBs, G2/M arrest, and apoptosis in CD44^+^ and CD44^−^ cells and, hence, could overcome the resistance seen for cisplatin. The low-energy Auger electrons induced DNA damage which is complex, specifically, due to clustered base damage proximal to the DSBs [27,28,48]. The combination of DNA DSBs and clustered damage challenges the repair machinery of the targeted cells, and ^125^I-induced DNA damage has previously been shown to be poorly repaired [28,49]. The resistance of CSCs relies on several protective mechanisms [12,13,14,15], e.g., DNA repair is higher in CSCs mainly due to the activation of the checkpoints. Checkpoint activation leads to cell cycle arrest and the attempted repair of damage. The uniform level of DNA DSBs by [^125^I]I-UdR in CD44^−^ and CD44^+^ cells were supported by similar levels of G2/M arrest irrespective of CD44 expression. Unrepairable Auger-induced DNA damage can lead to cell death by several pathways, including apoptosis and mitotic catastrophe [50,51]. However, we only tested for apoptosis and found no difference between the sorted populations. Interestingly, Auger electrons appeared equally effective in the sorted populations, which indicates that Auger electrons would also be able to overcome the plasticity of CSCs, i.e., the ability to switch between non-CSCs and CSCs [50].

Limitations to the study include that the magnetic sorting did not lead to pure sub-populations, especially for LUC13, making it more difficult to compare CD44^−^ and CD44^+^ subpopulations and even the first cell division could lead to heterogeneity in the subpopulations. We are also aware that the sorting process could affect the cells, e.g., Sutermaster et al. found that cell viability after magnetic sorting was reduced to 90% [40], and we cannot exclude other effects of the sorting. We focused on CD44, but other markers should also be tested, e.g., CD90 and CD133. To investigate if Auger-electrons could overcome cisplatin resistance, it would be interesting to develop cisplatin-resistant cells and analyze their response to Auger electrons. Strengths include that we tested patient-derived cells and that the cells were grown as tumorspheres to preserve homology to the parent tumor [31,52].

In conclusion, the CD44^+^ lung cancer cells were more resistant to cisplatin than the CD44^−^ cells. However, [^125^I]I-UdR was equally effective in CD44^−^ and CD44^+^ cells, indicating that the Auger electrons could overcome the resistance of CD44^+^ cells.

## 4. Materials and Methods

### 4.1. Establishment of Primary Cell Cultures

**Figure 9:**
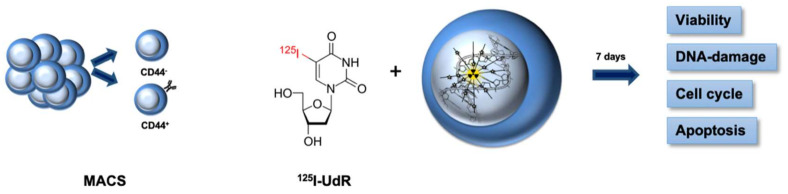
LUC10 and LUC13 were grown as tumorspheres. On the day of sorting, the tumorspheres were trypsinized and incubated with CD44 microbeads. The sorted cells were incubated with the Auger electron-emitting thymidine analog [^125^I]I-UdR for seven days, whereafter the viability, DNA damage, cell cycle, and apoptosis were analyzed.

Tumorspheres were established from NSCLC tissue surgically removed from lung cancer patients at Odense University Hospital. The Regional Ethics Committee of the region of Southern Denmark approved the protocol (S-20140170). As described in [33], the primary cells were prepared and grown in non-adherent flasks in serum-free medium at 37 °C in a humidified atmosphere with 5% CO_2_. The serum-free medium consisted of DMEM/F12 nutrient mix, Glutamax^TM^ supplemented with 1% penicillin/streptomycin, 1% B27, 20 ng/mL epidermal growth factor (all from Thermo Fisher Scientific, Roskilde, Denmark, Roskilde, Denmark), and 20 ng/mL basic fibroblast growth factor (Peprotech, Stockholm, Sweden)). A graphic overview of the experiments are presented in Figure 9.

### 4.2. Magnetic-Activated Cell Sorting (MACS)

Briefly, spheres were trypsinized, and cells were filtered through a 40 µM cell strainer to obtain single cells. One million cells were resuspended in 800 µL MACS buffer (0.5% bovine serum albumin (BSA), 2 mM EDTA in PBS) and incubated with 20 µL CD44 microbeads (Miltenyi Biotec, Lund, Sweden) for 15 min at 4 °C with gentle agitation. Cells were washed in MACS buffer, resuspended in 1 mL MACS buffer, and applied to an LS column (Miltenyi Biotec). The flow-through was collected as the CD44^−^ cells. The column was washed three times with 3 mL MACS buffer and removed from the separator, and the CD44^+^ cells were flushed out with 5 mL MACS buffer. The two sorted cell populations were washed twice in PBS, resuspended in serum-free medium, and used for subsequent experiments.

### 4.3. Sphere Formation Assay

The freshly sorted cells were diluted to 20 cells/mL, and 50 µL were transferred to each well in non-adherent 96-well plates. Wells containing only one cell were validated by microscopy and included in the experiment. Twice a week, 50 µL serum-free medium was added. After 21 days, the number of wells with a sphere was determined microscopically (Leica DMIL LED; Leica Microsystems, Wetzlar, Germany).

### 4.4. Quantitative PCR (qPCR)

Total RNA was purified from the freshly sorted cells using the RNeasy^®^ PLUS mini kit (Qiagen, Hilden, Germany) according to the manufacturer’s protocol. The RNA yield was measured using the Qubit 4 Fluorometer (Thermo Fisher Scientific, Roskilde, Denmark). RNA was reverse-transcribed using the RevertAid Minus first-strand cDNA synthesis kit and oligo(dT) primers (both from Thermo Fisher Scientific, Roskilde, Denmark) according to the manufacturer’s protocol. For each qPCR reaction, 5.5 ul TaqMan Fast Advanced Master Mix (Thermo Fisher Scientific, Roskilde, Denmark), 0.5 μL Taqman assay SOX2 (Hs01053049_s1), Nanog homeobox (NANOG; Hs04260366_g1), Pou class 5 homeobox 1 (POU5F1; Hs0099632_g1) and CD44 (Hs01075864_m1) (Thermo Fisher Scientific, Roskilde, Denmark) were mixed. Next, qPCR cycling was performed on a Quantstudio 3 (Applied Biosystems, Roskilde, Denmark) with a total of 20 ng cDNA per reaction as follows: 50 °C for 2 min and 95 °C for 2 min, and then 40 cycles of 95 °C for 1 s and 60 °C for 20 s. All reactions were performed in triplicate and normalized to hypoxanthine phosphoribosyltransferase 1 (HPRT1; Hs02800695_m1) and glyceraldehyde-3-phosphate dehydrogenase (GAPDH; Hs99999905_m1). Initially, four candidate reference genes (HPRT1, GAPDH, RPLP0, and ACTB) were tested, and HPRT1 and GAPDH were the most stable using the web-based analysis tool RefFinder [53]. Relative quantification was performed using the ∆∆C_q_ method [54].

### 4.5. Doubling Time (DT)

The freshly sorted cells were seeded at 5 × 10^4^ cells/mL in non-adherent 24-well plates and counted every second day. The DT was calculated as DT = *t*(log2)/(logN_t_ − logN_0_), where *t* was the culture time, and N_0_ and N_t_ were the initial and final cell numbers after seeding, respectively.

### 4.6. Proliferation and CD44 Expression

The freshly sorted cells were seeded in non-adherent 24-well plates (1 × 10^5^) and incubated with 10 µM 5-ethynyl-2′-deoxyuridine (EdU; MilliporeSigma, Copenhagen, Denmark) for 24 h. Subsequently, the cells were fixed with 3.7% formaldehyde for 15 min and permeabilized in 0.5% Triton X-100 in PBS for 20 min. The cells were stained using the Click-iT EdU 488 Proliferation Kit (MilliporeSigma, Copenhagen, Denmark) following the manufacturer’s protocol. Next, cells were washed twice in 3% BSA/PBS and blocked for 1 h in 2% BSA/2% FBS in PBS. Cells were incubated in the dark for 20 min with 150 µL CD44-PE (1:5000) (Miltenyi Biotec) and counterstained with 10 µg/mL DAPI (MilliporeSigma, Copenhagen, Denmark) for 10 min. Cells (200–600) were analyzed using a Leica DM 2000 LED microscope (Leica Microsystems, Copenhagen, Denmark). Quantification was performed manually using the ‘cell counter plugin’ in ImageJ version 1.50i (National Institute of Health, Bethesda, MD, USA).

### 4.7. Cellular Uptake and DNA Incorporation of [^125^I]I-UdR

Forty thousand freshly sorted cells were incubated with 18.5 kBq/mL [^125^I]I-UdR (prepared as in [36] in 1 mL serum-free medium for 1, 4, and 7 h in non-adherent 24-well plates). At each time point, the cells were washed twice with 400 µL cold PBS and 400 µL 5% trichloroacetic acid (TCA). The pelleted DNA was solubilized in 500 µL NaOH. The radioactivity in the TCA fractions and collected DNA was determined in a 2470 Wizard Automatic Gamma Counter (Perkin Elmer, Skovlunde, Denmark). Cellular uptake was given by the sum of radioactivity in the TCA fractions and collected DNA relative to added radioactivity (% of injected dose/well). The DNA incorporation was calculated as the percentage of radioactivity in the DNA relative to added radioactivity (% of injected dose/well).

### 4.8. Viability Assay

One thousand freshly sorted cells were seeded in quadruplicate in non-adherent 96-well plates in 50 µL serum-free medium with 0.1–6.0 kBq/mL [^125^I]I-UdR or 0.1–50 μM cisplatin (MilliporeSigma, Copenhagen, Denmark) in 50 µL media. Non-radioactive but chemically identical [^127^I]I-UdR (24 pg/mL; MilliporeSigma, Copenhagen, Denmark) corresponding to 6 kBq/mL mass concentration was included as a control. On day seven (day three for cisplatin), the cell viability was evaluated by adding 13 µL CellTiter-Blue (Promega, Nacka, Sweden) to each well. Fluorescence was measured at 520nm excitation/580–640nm emission in a GloMax Explorer (Promega, Nacka, Sweden).

### 4.9. DNA Damage, Cell Cycle, and Apoptosis

The freshly sorted cells (1 × 10^5^) were seeded in non-adherent 24-well plates in 1 mL serum-free medium. Cells were incubated with 2.5 kBq/mL [^125^I]I-UdR for 7 days to evaluate DNA damage, cell cycle, and apoptosis. DNA damage and apoptosis were also analyzed in freshly sorted cells incubated with 10 μM cisplatin for 2 and 72 h, respectively. At the indicated time points, cells were trypsinized and counted using the MUSE Count & Viability Reagent (Luminex, ´s-Hertogenbosch, The Netherlands) before being used for DNA damage, cell cycle, or apoptosis analyses.

For DNA damage, fifty thousand cells were analyzed using the MUSE H2AX Activation kit (Luminex). Briefly, cells were resuspended in 50 µL 1× Assay buffer and 50 µL fixation reagent for 5 min on ice. Subsequently, the cells were permeabilized in a 50 µL ice-cold permeabilization reagent for 5 min on ice. Cells were incubated in 50 µL 1× Assay buffer containing 1 µL anti-H2A.X (Luminex) and 1 µL anti-phospho-histone H2A.X (Luminex) at room temperature in the dark for 30 min. Cells were washed with 100 µL 1× Assay buffer, resuspended in 200 µL 1× Assay buffer, and analyzed on the Guava MUSE Cell Analyzer (Luminex).

Cells were fixed in 70% ethanol at −20 °C overnight for cell cycle analysis. After fixation, cells were washed in PBS and resuspended in 100 µL cell cycle reagent mix (20 µg/mL propidium iodide and 10 mg/mL RNase A, both from MilliporeSigma (Copenhagen, Denmark) and incubated for 30 min in the dark at room temperature. Next, cells were washed in PBS and loaded into an A8 cassette (Chemometec, Alleroed, Denmark). Cell cycle distribution was measured by image cytometry in the Nucleocounter NC-3000 (Chemometec).

For apoptosis analysis, fifty thousand cells were resuspended in 50 µL 1% BSA/PBS and mixed with 50 µL MUSE Annexin V and Dead Reagent (Luminex). The samples were incubated at room temperature in the dark for 20 min and analyzed on the Guava MUSE Cell Analyzer.

### 4.10. Statistical Analysis

Experiments were performed as three independent replicates, and descriptive statistics for quantitative measurements comprised the mean ± standard deviation. The sphere formation assay was statistically assessed using the Student *t*-test. Two-way ANOVA was used to compare the means of proliferation and CD44 expression (correction for multiple comparisons: Ŝidák). Two-way ANOVA was applied (correction for multiple comparisons: Tukey) to compare the means of the relative expression of genes, doubling time, uptake, and the incorporation of [^125^I]I-UdR, viability, DNA damage activity, cell cycle, and apoptosis. *p* < 0.05 was considered as a threshold of statistical significance. Statistical tests were performed using GraphPad Prism version 9.0 (GraphPad Software, San Diego, CA, USA).

## Data Availability

The datasets used and/or analyzed in the current study are available from the corresponding author upon request.

## References

[1] Sung H., Ferlay J., Siegel R.L., Laversanne M., Soerjomataram I., Jemal A., Bray F. (2021). Global Cancer Statistics 2020: GLOBOCAN Estimates of Incidence and Mortality Worldwide for 36 Cancers in 185 Countries. CA Cancer J. Clin..

[2] Travis W.D. (2011). Pathology of lung cancer. Clin. Chest Med..

[3] Heng W.S., Gosens R., Kruyt F.A.E. (2019). Lung cancer stem cells: Origin, features, maintenance mechanisms and therapeutic targeting. Biochem. Pharmacol..

[4] MacDonagh L., Gray S.G., Breen E., Cuffe S., Finn S.P., O’Byrne K.J., Barr M.P. (2016). Lung cancer stem cells: The root of resistance. Cancer Lett..

[5] Leonetti A., Sharma S., Minari R., Perego P., Giovannetti E., Tiseo M. (2019). Resistance mechanisms to osimertinib in EGFR-mutated non-small cell lung cancer. Br. J. Cancer.

[6] Wu H., Mu X., Liu L., Wu H., Hu X., Chen L., Liu J., Mu Y., Yuan F., Liu W. (2020). Bone marrow mesenchymal stem cells-derived exosomal microRNA-193a reduces cisplatin resistance of non-small cell lung cancer cells via targeting LRRC1. Cell Death Dis..

[7] Wang L., Liu X., Ren Y., Zhang J., Chen J., Zhou W., Guo W., Wang X., Chen H., Li M. (2017). Cisplatin-enriching cancer stem cells confer multidrug resistance in non-small cell lung cancer via enhancing TRIB1/HDAC activity. Cell Death Dis..

[8] Phi L.T.H., Sari I.N., Yang Y.G., Lee S.H., Jun N., Kim K.S., Lee Y.K., Kwon H.Y. (2018). Cancer Stem Cells (CSCs) in Drug Resistance and their Therapeutic Implications in Cancer Treatment. Stem Cells Int..

[9] Arnold C.R., Mangesius J., Skvortsova I., Ganswindt U. (2020). The Role of Cancer Stem Cells in Radiation Resistance. Front. Oncol..

[10] Abbaszadegan M.R., Bagheri V., Razavi M.S., Momtazi A.A., Sahebkar A., Gholamin M. (2017). Isolation, identification, and characterization of cancer stem cells: A review. J. Cell Physiol..

[11] Atashzar M.R., Baharlou R., Karami J., Abdollahi H., Rezaei R., Pourramezan F., Zoljalali Moghaddam S.H. (2020). Cancer stem cells: A review from origin to therapeutic implications. J. Cell Physiol..

[12] Zhou W., Sun M., Li G.H., Wu Y.Z., Wang Y., Jin F., Zhang Y.Y., Yang L., Wang D.L. (2013). Activation of the phosphorylation of ATM contributes to radioresistance of glioma stem cells. Oncol. Rep..

[13] Phillips T.M., McBride W.H., Pajonk F. (2006). The response of CD24(-/low)/CD44+ breast cancer-initiating cells to radiation. J. Natl. Cancer Inst..

[14] Bao S., Wu Q., McLendon R.E., Hao Y., Shi Q., Hjelmeland A.B., Dewhirst M.W., Bigner D.D., Rich J.N. (2006). Glioma stem cells promote radioresistance by preferential activation of the DNA damage response. Nature.

[15] Diehn M., Cho R.W., Lobo N.A., Kalisky T., Dorie M.J., Kulp A.N., Qian D., Lam J.S., Ailles L.E., Wong M. (2009). Association of reactive oxygen species levels and radioresistance in cancer stem cells. Nature.

[16] Prabavathy D., Swarnalatha Y., Ramadoss N. (2018). Lung cancer stem cells-origin, characteristics and therapy. Stem Cell Investig..

[17] Leung E.L., Fiscus R.R., Tung J.W., Tin V.P., Cheng L.C., Sihoe A.D., Fink L.M., Ma Y., Wong M.P. (2010). Non-small cell lung cancer cells expressing CD44 are enriched for stem cell-like properties. PLoS ONE.

[18] Xu H., Niu M., Yuan X., Wu K., Liu A. (2020). CD44 as a tumor biomarker and therapeutic target. Exp. Hematol. Oncol..

[19] Luo Z., Wu R.R., Lv L., Li P., Zhang L.Y., Hao Q.L., Li W. (2014). Prognostic value of CD44 expression in non-small cell lung cancer: A systematic review. Int. J. Clin. Exp. Pathol..

[20] Wang Y.Y., Vadhan A., Chen P.H., Lee Y.L., Chao C.Y., Cheng K.H., Chang Y.C., Hu S.C., Yuan S.F. (2021). CD44 Promotes Lung Cancer Cell Metastasis through ERK-ZEB1 Signaling. Cancers.

[21] Chen K., Huang Y.H., Chen J.L. (2013). Understanding and targeting cancer stem cells: Therapeutic implications and challenges. Acta Pharmacol. Sin..

[22] Chan C., Fonge H., Lam K., Reilly R.M. (2020). Effectiveness and normal tissue toxicity of Auger electron (AE) radioimmunotherapy (RIT) with [(111)In]In-Bn-DTPA-nimotuzumab in mice with triple-negative or trastuzumab-resistant human breast cancer xenografts that overexpress EGFR. Nucl. Med. Biol..

[23] Morgenroth A., Vogg A.T., Zlatopolskiy B.D., Siluschek M., Oedekoven C., Mottaghy F.M. (2014). Breaking the invulnerability of cancer stem cells: Two-step strategy to kill the stem-like cell subpopulation of multiple myeloma. Mol. Cancer Ther..

[24] Pirovano G., Jannetti S.A., Carter L.M., Sadique A., Kossatz S., Guru N., Demetrio De Souza Franca P., Maeda M., Zeglis B.M., Lewis J.S. (2020). Targeted Brain Tumor Radiotherapy Using an Auger Emitter. Clin. Cancer Res..

[25] Morgenroth A., Vogg A.T., Ermert K., Zlatopolskiy B., Mottaghy F.M. (2014). Hedgehog signaling sensitizes glioma stem cells to endogenous nano-irradiation. Oncotarget.

[26] Kassis A.I. (2004). The amazing world of auger electrons. Int. J. Radiat. Biol..

[27] Balagurumoorthy P., Chen K., Adelstein S.J., Kassis A.I. (2008). Auger electron-induced double-strand breaks depend on DNA topology. Radiat. Res..

[28] Schmitz S., Oskamp D., Pomplun E., Kriehuber R. (2015). Chromosome aberrations induced by the Auger electron emitter (125)I. Mutat. Res. Genet. Toxicol. Environ. Mutagen..

[29] Schulz A., Meyer F., Dubrovska A., Borgmann K. (2019). Cancer Stem Cells and Radioresistance: DNA Repair and Beyond. Cancers.

[30] Eramo A., Lotti F., Sette G., Pilozzi E., Biffoni M., Di Virgilio A., Conticello C., Ruco L., Peschle C., De Maria R. (2008). Identification and expansion of the tumorigenic lung cancer stem cell population. Cell Death Differ..

[31] Herreros-Pomares A., de-Maya-Girones J.D., Calabuig-Farinas S., Lucas R., Martinez A., Pardo-Sanchez J.M., Alonso S., Blasco A., Guijarro R., Martorell M. (2019). Lung tumorspheres reveal cancer stem cell-like properties and a score with prognostic impact in resected non-small-cell lung cancer. Cell Death Dis..

[32] Nishino M., Ozaki M., Hegab A.E., Hamamoto J., Kagawa S., Arai D., Yasuda H., Naoki K., Soejima K., Saya H. (2017). Variant CD44 expression is enriching for a cell population with cancer stem cell-like characteristics in human lung adenocarcinoma. J. Cancer.

[33] Madsen K.L., Langkjaer N., Gerke O., Hoilund-Carlsen P.F., Olsen B.B. (2022). Establishment of patientderived lung tumorspheres and their response to internal irradiation by Auger electrons. Int. J. Oncol..

[34] Rogakou E.P., Pilch D.R., Orr A.H., Ivanova V.S., Bonner W.M. (1998). DNA double-stranded breaks induce histone H2AX phosphorylation on serine 139. J. Biol. Chem..

[35] Thisgaard H., Halle B., Aaberg-Jessen C., Olsen B.B., Therkelsen A.S., Dam J.H., Langkjaer N., Munthe S., Nagren K., Hoilund-Carlsen P.F. (2016). Highly Effective Auger-Electron Therapy in an Orthotopic Glioblastoma Xenograft Model using Convection-Enhanced Delivery. Theranostics.

[36] Madsen K.L., Therkelsen A.S.N., Langkjaer N., Olsen B.B., Thisgaard H. (2021). Auger electron therapy of glioblastoma using [(125)I]5-iodo-2’-deoxyuridine and concomitant chemotherapy—Evaluation of a potential treatment strategy. Nucl. Med. Biol..

[37] Pustovalova M., Blokhina T., Alhaddad L., Chigasova A., Chuprov-Netochin R., Veviorskiy A., Filkov G., Osipov A.N., Leonov S. (2022). CD44+ and CD133+ Non-Small Cell Lung Cancer Cells Exhibit DNA Damage Response Pathways and Dormant Polyploid Giant Cancer Cell Enrichment Relating to Their p53 Status. Int. J. Mol. Sci..

[38] Zhou Y., Chen H., Li H., Wu Y. (2017). 3D culture increases pluripotent gene expression in mesenchymal stem cells through relaxation of cytoskeleton tension. J. Cell Mol. Med..

[39] Ko Y.H., Won H.S., Jeon E.K., Hong S.H., Roh S.Y., Hong Y.S., Byun J.H., Jung C.K., Kang J.H. (2011). Prognostic significance of CD44s expression in resected non-small cell lung cancer. BMC Cancer.

[40] Sutermaster B.A., Darling E.M. (2019). Considerations for high-yield, high-throughput cell enrichment: Fluorescence versus magnetic sorting. Sci. Rep..

[41] Di Stefano C., Grazioli P., Fontanella R.A., De Cesaris P., D’Amore A., Regno M., Starace D., Padula F., Fiori M.E., Canipari R. (2018). Stem-like and highly invasive prostate cancer cells expressing CD44v8-10 marker originate from CD44-negative cells. Oncotarget.

[42] Hu B., Ma Y., Yang Y., Zhang L., Han H., Chen J. (2018). CD44 promotes cell proliferation in non-small cell lung cancer. Oncol. Lett..

[43] Alowaidi F., Hashimi S.M., Alqurashi N., Alhulais R., Ivanovski S., Bellette B., Meedenyia A., Lam A., Wood S. (2018). Assessing stemness and proliferation properties of the newly established colon cancer ‘stem’ cell line, CSC480 and novel approaches to identify dormant cancer cells. Oncol. Rep..

[44] Yin J., Zhang H., Wu X., Zhang Y., Li J., Shen J., Zhao Y., Xiao Z., Lu L., Huang C. (2020). CD44 inhibition attenuates EGFR signaling and enhances cisplatin sensitivity in human EGFR wildtype non-small-cell lung cancer cells. Int. J. Mol. Med..

[45] Barr M.P., Gray S.G., Hoffmann A.C., Hilger R.A., Thomale J., O’Flaherty J.D., Fennell D.A., Richard D., O’Leary J.J., O’Byrne K.J. (2013). Generation and characterisation of cisplatin-resistant non-small cell lung cancer cell lines displaying a stem-like signature. PLoS ONE.

[46] Jung Y., Lippard S.J. (2007). Direct cellular responses to platinum-induced DNA damage. Chem. Rev..

[47] Goto S., Kawabata T., Li T.S. (2020). Enhanced Expression of ABCB1 and Nrf2 in CD133-Positive Cancer Stem Cells Associates with Doxorubicin Resistance. Stem Cells Int..

[48] Raisali G., Mirzakhanian L., Masoudi S.F., Semsarha F. (2013). Calculation of DNA strand breaks due to direct and indirect effects of Auger electrons from incorporated 123I and 125I radionuclides using the Geant4 computer code. Int. J. Radiat. Biol..

[49] Datta K., Neumann R.D., Winters T.A. (2005). Characterization of a complex I-125-induced DNA double-strand break: Implications for repair. Int. J. Radiat. Biol.

[50] Haefliger P., Agorastos N., Renard A., Giambonini-Brugnoli G., Marty C., Alberto R. (2005). Cell uptake and radiotoxicity studies of an nuclear localization signal peptide-intercalator conjugate labeled with [99mTc(CO)3]+. Bioconjug. Chem..

[51] Urashima T., Nagasawa H., Wang K., Adelstein S.J., Little J.B., Kassis A.I. (2006). Induction of apoptosis in human tumor cells after exposure to Auger electrons: Comparison with gamma-ray exposure. Nucl. Med. Biol..

[52] Lee J., Kotliarova S., Kotliarov Y., Li A., Su Q., Donin N.M., Pastorino S., Purow B.W., Christopher N., Zhang W. (2006). Tumor stem cells derived from glioblastomas cultured in bFGF and EGF more closely mirror the phenotype and genotype of primary tumors than do serum-cultured cell lines. Cancer Cell.

[53] Xie F., Xiao P., Chen D., Xu L., Zhang B. (2012). miRDeepFinder: A miRNA analysis tool for deep sequencing of plant small RNAs. Plant. Mol. Biol..

[54] Livak K.J., Schmittgen T.D. (2001). Analysis of relative gene expression data using real-time quantitative PCR and the 2(-Delta Delta C(T)) Method. Methods.

